# Symmetrical retrograde actin flow in the actin fusion structure is involved in osteoclast fusion

**DOI:** 10.1242/bio.025460

**Published:** 2017-07-15

**Authors:** Jiro Takito, Hirotada Otsuka, Satoshi Inoue, Tsubasa Kawashima, Masanori Nakamura

**Affiliations:** 1 Department of Oral Anatomy and Developmental Biology, School of Dentistry, Showa University, 1-5-8 Hatanodai, Shinagawa, Tokyo 142-8555, Japan; 2 Department of Paediatric Dentistry, School of Dentistry, Showa University, 1-5-8 Hatanodai, Shinagawa, Tokyo 142-8555, Japan

**Keywords:** Actin, Cell fusion, Non-muscle myosin IIA, Osteoclast, Podosome

## Abstract

The aim of this study was to elucidate the role of the zipper-like structure (ZLS), a podosome-related structure that transiently appears at the cell contact zone, in osteoclast fusion. Live-cell imaging of osteoclasts derived from RAW264.7 cells transfected with EGFP-actin revealed consistent symmetrical retrograde actin flow in the ZLS, but not in the podosome cluster, the podosome ring or the podosome belt. Confocal imaging showed that the distributions of F-actin, vinculin, paxillin and zyxin in the ZLS were different from those in the podosome belt. Thick actin filament bundles running outside the ZLS appeared to recruit non-muscle myosin IIA. The F-actin-rich domain of the ZLS contained actin-related protein 2/3 complex (Arp2/3). Inhibition of Arp2/3 activity disorganized the ZLS, disrupted actin flow, deteriorated cell-cell adhesion and inhibited osteoclast hypermultinucleation. In contrast, ML-7, an inhibitor of myosin light chain kinase, had little effect on the structure of ZLS and promoted osteoclast hypermultinucleation. These results reveal a link between actin flow in the ZLS and osteoclast fusion. Osteoclast fusion was promoted by branched actin elongation and negatively regulated by actomyosin contraction.

## Abstract (precis)

**Summary:** Multinucleated osteoclasts form a podosome-derived fusion structure during cell fusion. Juxtaposition of fusion partner cells is probably maintained via force generated by symmetrical retrograde actin flow in the fusion structure.

## INTRODUCTION

Osteoclasts are multinucleated bone-resorbing cells that differentiate from monocyte-lineage mononuclear precursors (Boyle et al., 2003). Osteoclast multinucleation promotes efficient bone resorption (Yagi et al., 2005). Many membrane proteins that stimulate osteoclast fusion have been discovered, such as ATP6v0d2 (Lee et al., 2006), CD9 (Ishii et al., 2006), DC-STAMP (Kukita et al., 2004; Yagi et al., 2005) and syncytin-1 (Søe et al., 2011). However, the mechanisms by which these proteins promote osteoclast fusion remain unclear. Heterogeneity is one of the difficulties that hamper our understanding of the mechanisms regulating osteoclast fusion. Fusion can occur between two cells of the same type (mononuclear or multinucleated), or between a mononuclear cell and a multinucleated cell. Moreover, various types of actin structure connect the two cells before and during membrane fusion (Oikawa et al., 2012; Song et al., 2014; Suzuki et al., 2002; Takahashi et al., 2013; Takito et al., 2012; Wang et al., 2015). Two research groups have monitored osteoclast fusion events by live-cell imaging, and grouped this complex phenomenon in simpler classes. Søe et al. (Søe et al., 2015) proposed three categories of human osteoclast fusion based on the morphology of the site in which the fusion partners are connected: a broad contact area (40%), a phagocytic cup (45%), and others (15%). Another group (Fiorino and Harrison, 2016) categorized murine osteoclast fusion based on the fusion site location: at the leading lamella (64%), at active membrane extensions/protrusions (23%) and in other locations (14%). Such categorization may help to delineate the sequential events occurring during osteoclast fusion. We previously reported a novel type of actin superstructure, named the zipper-like structure (ZLS), in the RAW 264.7 cell line and in mouse osteoclasts *in vivo* (Takito et al., 2012). The RAW 264.7 cell line has been widely used as an *in vitro* model of many aspects of osteoclast differentiation, including osteoclast fusion (Hsu et al., 1999). According to the classification described above, the ZLS is a main fusion structure that appears at the lamella, and creates a broad contact surface for the fusion between multinucleated cells.

Osteoclasts attach to and resorb bone via the sealing zone, another actin superstructure (Boyce et al., 1992; Miyauchi et al., 1990). During osteoclast differentiation on glass, podosome-related structures develop at their ventral plasma membrane (Destaing et al., 2003; Ory et al., 2008). The cells first develop a podosome cluster, which then grows into a small ring-like structure, the podosome ring. One osteoclast often has several podosome rings. The podosome ring develops into a large ring running along the cell periphery, known as the podosome belt (an *in vitro* equivalent of the sealing zone), which transforms into the ZLS at the contact site between two osteoclasts (Takito and Nakamura, 2012). This transformation is most likely stimulated by cell contact. The ZLS spans both cells and is involved in cell-cell interactions, whereas other podosome-related superstructures appear in single cells and are engaged in cell-extracellular matrix interactions. These divergent actin structures in osteoclasts evolve from the podosome, a dot-like actin-rich structure consisting of a central actin core surrounded by an adhesion domain (Destaing et al., 2003; Linder and Wiesner, 2014). Podosomes attach to the matrix via the adhesion domain, which contains integrin β3, vinculin, talin, paxillin and zyxin. Neighboring podosomes are interconnected via F-actin cables. The formation and maintenance of the actin core depends on the actin polymerizing factors actin-related protein 2/3 complex (Arp2/3) (Kaverina et al., 2003) and cortactin (Mizutani et al., 2002), as well as depolymerizing factors. Directed polymerization of actin filaments in the podosome core is thought to generate protrusive forces against the matrix (Luxenburg et al., 2012). It is proposed that these protrusion forces are involved in cancer cell invasion (Gawden-Bone et al., 2010) and osteoclast fusion (Oikawa et al., 2012; Shin et al., 2014).

The aim of this study was to elucidate the role of the ZLS in cell-cell interactions during osteoclast fusion. We found that EGFP-actin flows symmetrically throughout the ZLS. The actin flow may generate forces that facilitate the juxtaposition of the multinucleated partner cells before and during cell fusion.

## RESULTS AND DISCUSSION

### Symmetrical retrograde actin flow in the ZLS

To gather information on the dynamics of the ZLS, we introduced EGFP-actin into RAW 264.7 cells for live-cell imaging. We have already recorded the ZLS actin dynamics at 1 min intervals (Takito and Nakamura, 2012; Takito et al., 2012), but because recent experiments revealed that the oscillation period of the actin core is shorter (40 s) in human macrophage podosomes (Labernadie et al., 2014), we reexamined the actin dynamics in osteoclasts at 4 s intervals and at higher magnifications in this study.

**Fig. 1.**
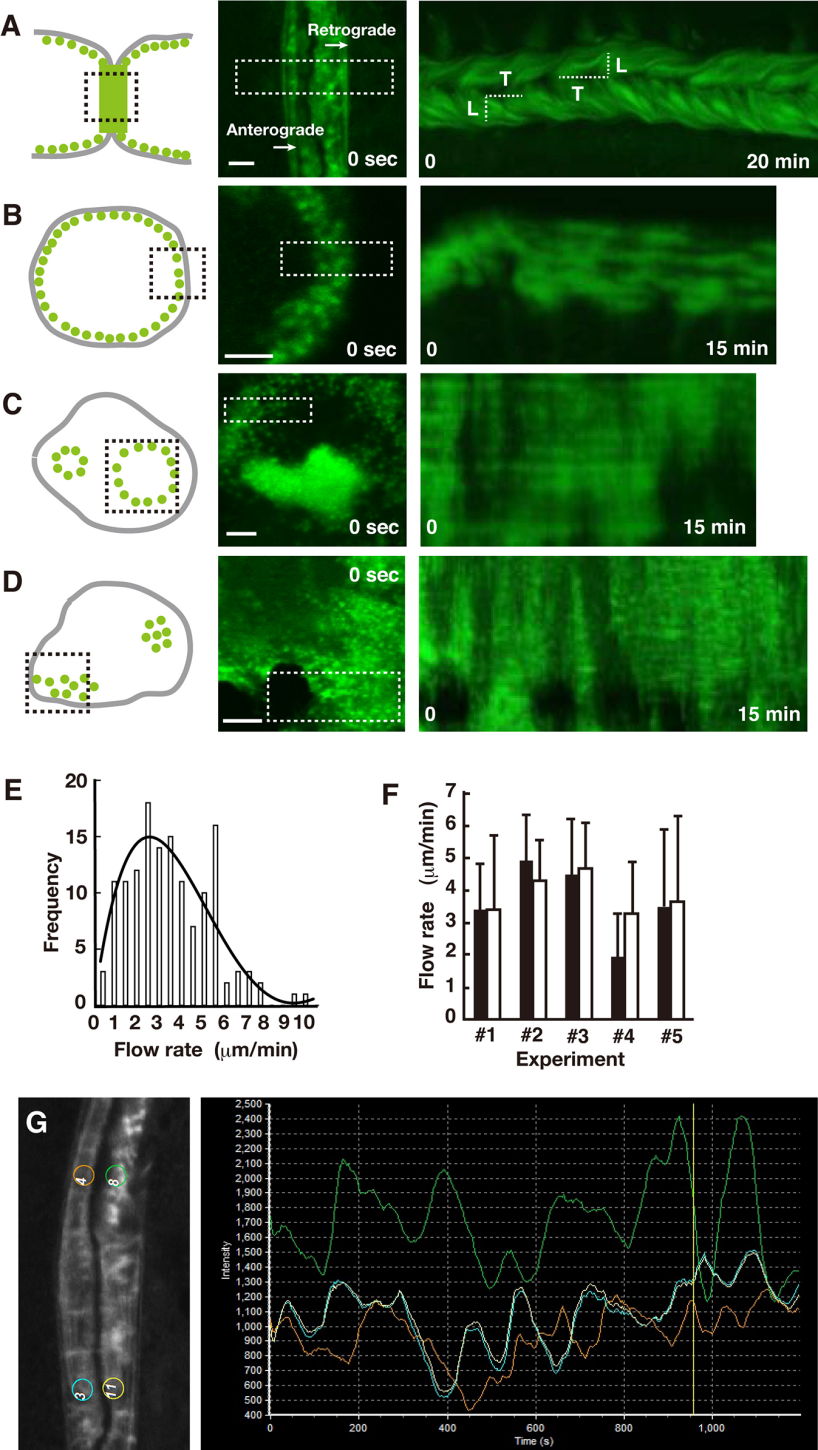
**Characteristics of actin dynamics in the ZLS.** (A-D) RAW 264.7 cells were transfected with EGFP-actin and used for live-cell imaging. Schematics (left panel), confocal images at time 0 (center panel), and the corresponding kymographs (right panel) of the ZLS (A), the podosome belt (B), the podosome ring (C), and the podosome cluster (D) are shown. In the left panel, each actin structure in an osteoclast (gray solid line) is depicted by the arrangement of the actin cores (green dots). Time series confocal images were acquired in the area indicated by the black dotted line box. White dotted line boxes in the center panel indicate the area selected to generate the kymographs. The actin flow rate was determined by dividing the distance of EGFP-actin movement (L) by the elapsed time (T) in a kymograph. Scale bars: 5 µm. (E) Histogram of retrograde actin flow rate (*n*=140). The distribution of the flow rates was fitted with a quartic curve. (F) Comparison of the actin flow rate in a given cell (□) and the opposing cell (▪) in the ZLS. Flow rate was determined from 10 different actin flows from a single kymograph. Data are mean±s.d. The flow rate in a given cell was not significantly different from that of the opposing cell in five ZLS (*P*>0.05, paired *t*-test). (G) Oscillation of EGFP-actin fluorescence in the ZLS. Left panel, four regions of interest (ROIs) were placed in symmetrical positions between the two cells and in a distant location in the same cell. The number of ROIs was arbitrary. Right panel, time-lapse recordings of the EGFP fluorescence from the four ROIs.

The EGFP-actin signals revealed three types of actin structure in the ZLS: podosome-like dots, stripes that ran perpendicular to the cell contact surface, and faint clouds. Time-lapse imaging revealed the retrograde bulk flow of EGFP-actin and the concomitant movement of the actin dots and stripes in the ZLS (Fig. 1A; Movie 1). EGFP kymographs generated from the time-lapse images indicate that the retrograde actin flow is robust and consistent during the recording time (*n*=23), and cannot be caused by the dissolution and re-formation of podosomes. Unlike in the ZLS, the EGFP-actin signals in the podosome belt (*n*=84) (Fig. 1B; Movie 2) and the podosome ring (*n*=24) (Fig. 1C; Movie 3) did not show such consistent dynamics. Directional bulk flow of actin in these structures was often observed for a short time. We also observed occasional flow of bulk EGFP-actin in the podosome cluster (*n*=42) (Fig. 1D; Movie 4). These results suggest that movement of EGFP-actin occurs frequently in the podosome cluster, the podosome ring, the podosome belt and the ZLS in osteoclasts. However, the ZLS was the only superstructure that supported consistent vectorial flow of actin. Ventral actin waves, known as traveling waves, are observed in various cells (Case and Waterman, 2011; Weiner et al., 2007). We also observed traveling waves in osteoclasts. To avoid confusion, actin flow, analyzed in this study, is defined as the movement of EGFP-actin within the area containing the podosomes, and traveling waves as the movement of EGFP-actin in the area containing no podosomes. The latter was distinguished from the former by its lower intensity in osteoclasts (Movie 2).

In the ZLS, movement of the podosome-like dots and elongation of the stripes from the cell edge to the cell interior were frequently observed. The flow rate of actin, estimated from the trajectory of EGFP-actin in the kymographs (Fig. 1A), was in the range of 0.5-10 µm/min, with a mean value of 3.32±1.94 µm/min (mean±s.d., *n*=140) (Fig. 1E). The flow rate distribution was approximated by the quartic curve, suggesting the heterogeneity of flowing actin structures in the ZLS. Fig. 1F shows that the flow rate of F-actin in one cell was not significantly different from that in the opposite cell connected by the ZLS. Thus, the two osteoclasts exhibited symmetrical retrograde actin flow at their contact site. We recorded the actin flow at 1.0 µm intervals along the Z-axis, and observed that it was uniform within a volume of 2.5 µm in height (data not shown).

Actin oscillation is one of the characteristics of the podosomes in the podosome belt of osteoclasts (Destaing et al., 2003). Similar to the podosome belt, all ZLS examined (11/11) showed EGFP-actin signal oscillation, which consistently lasted over 30 min, with a period of 1-3 min. Occasionally, we observed oscillation synchronization between two distant locations [regions of interest (ROI) 3 and 11 in Fig. 1G], further confirming the synchronization of actin flow between two cells (Fig. 1F).

### Static structure of F-actin in podosome-related structures

**Fig. 2.**
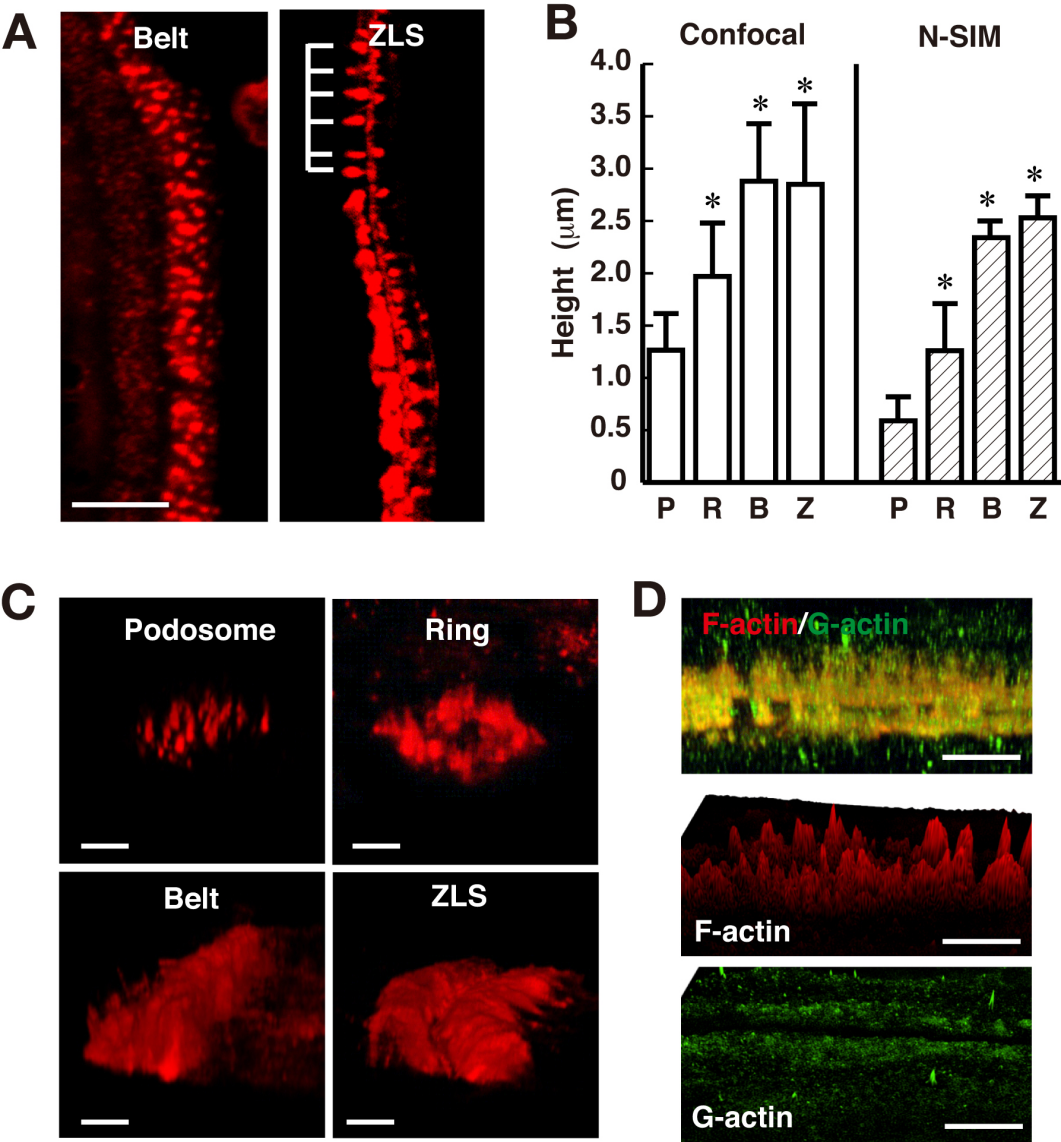
**Dimensions of F-actin structures in the ZLS.** Osteoclasts were stained with rhodamine-phalloidin. (A) Confocal images showing the podosome belt (left) and the ZLS (right). Note the repeated appearance of F-actin structures in the ZLS (indicated by a comb). Scale bar: 10 µm. (B) The height of the podosome in the podosome cluster (P), the podosome ring (R), the podosome belt (B), and the ZLS (Z) was determined from confocal (left) and N-SIM (right) images. The height was measured as described in the Materials and Methods. Confocal observations: podosome cluster, *n*=34; podosome ring, *n*=26; podosome belt, *n*=39; ZLS, *n*=33. N-SIM observations: podosome cluster, *n*=120; podosome ring, *n*=6; podosome belt, *n*=6; ZLS, *n*=5. Data are mean±s.d. *Significantly different from the podosome in the podosome cluster, *P*<0.01, paired *t*-test. (C) Tilted volume view of the podosome-related superstructures. Osteoclasts were stained with rhodamine-phalloidin for N-SIM. Scale bars: 2 µm. (D) Distribution of F- and G-actin in the ZLS. Cells were stained with rhodamine-phalloidin (red) and an anti-G-actin antibody (green) for N-SIM imaging. Upper panel, tilted volume view of the ZLS. Middle panel, surface intensity profiles of F-actin. Bottom panel, surface intensity profiles of G-actin. Scale bars: 5 µm.

The above findings on actin dynamics in the ZLS prompted us to elucidate the detailed arrangement of podosomal proteins by immunofluorescence. First, we determined the static structure of F-actin in the ZLS by staining fixed osteoclasts with rhodamine-phalloidin. Measurements in the projected confocal images showed that F-actin formed a band along the cell periphery with an average width and length of 8.37±2.0 µm (*n*=19) and 159±146 µm (*n*=36), respectively. As expected, the width value was twice that of the podosome belt (Saltel et al., 2008). The large variation in length may be related to the size of the contact surface between the two cells, suggesting a possible tangential repeat unit in the ZLS. Accordingly, F-actin often formed stripes with repeated peaks along the length of the ZLS (Fig. 2A). The distance between two peaks in the ZLS was 2.03±0.81 µm (*n*=92), whereas the interpodosomal distance in the podosome cluster was 1.53±0.57 µm (*n*=156). In osteoclasts cultured on bone, the interpodosomal distance in the sealing zone is smaller than that in the podosome cluster (Luxenburg et al., 2007). Therefore, the actin core density in the ZLS is lower than in the sealing zone.

The ZLS height determined by length measurements in the projected confocal microscopy images was 2.85±0.77 µm (Fig. 2C). A similar value was obtained for the height of the podosome belt (2.88±0.56 µm). However, the height of the podosome in the podosome cluster and podosome ring was 1.26±0.34 and 1.96±0.52 µm, respectively. Previous analysis by atomic force microscopy provided a value of 0.57±0.12 µm for the height of the podosome (Labernadie et al., 2010), suggesting that our values are probably overestimates, likely due to fluorescence scattering in confocal microscopy. To circumvent this problem, we used super-resolution microscopy (Fig. 2C). Indeed, Nikon structured illumination microscopy (N-SIM) resulted in a value of 0.59±0.25 µm for the height of the podosome in the podosome cluster (Fig. 2B). The mean height of F-actin structures in the podosome belt and the ZLS determined by N-SIM was 2.37±0.16 and 2.53±0.21 µm, respectively. These results suggest that the height of the F-actin structures in the podosome ring, the podosome belt and the ZLS is significantly larger than that of the podosomes in the podosome cluster.

We compared the distributions of G-actin and F-actin in the ZLS by immunofluorescence using N-SIM. The anti-β-actin antibody used did not recognize phalloidin-positive stress fibers and microplicae (Fig. S1), and thus appeared to be specific for G-actin. The distribution of G-actin overlapped with that of F-actin structures (Fig. 2D). The height of the G-actin band was 2.43±0.45 mm (*n*=4), which is comparable to the height of the F-actin structures. The surface intensity profiles indicate that G-actin was uniformly distributed along the length of the ZLS, while F-actin formed repeated peaks. These results are consistent with the idea that F-actin structures coexist with the G-actin band in which actin flow occurs.

### Polarized distribution of non-muscle myosin IIA in the ZLS

**Fig. 3.**
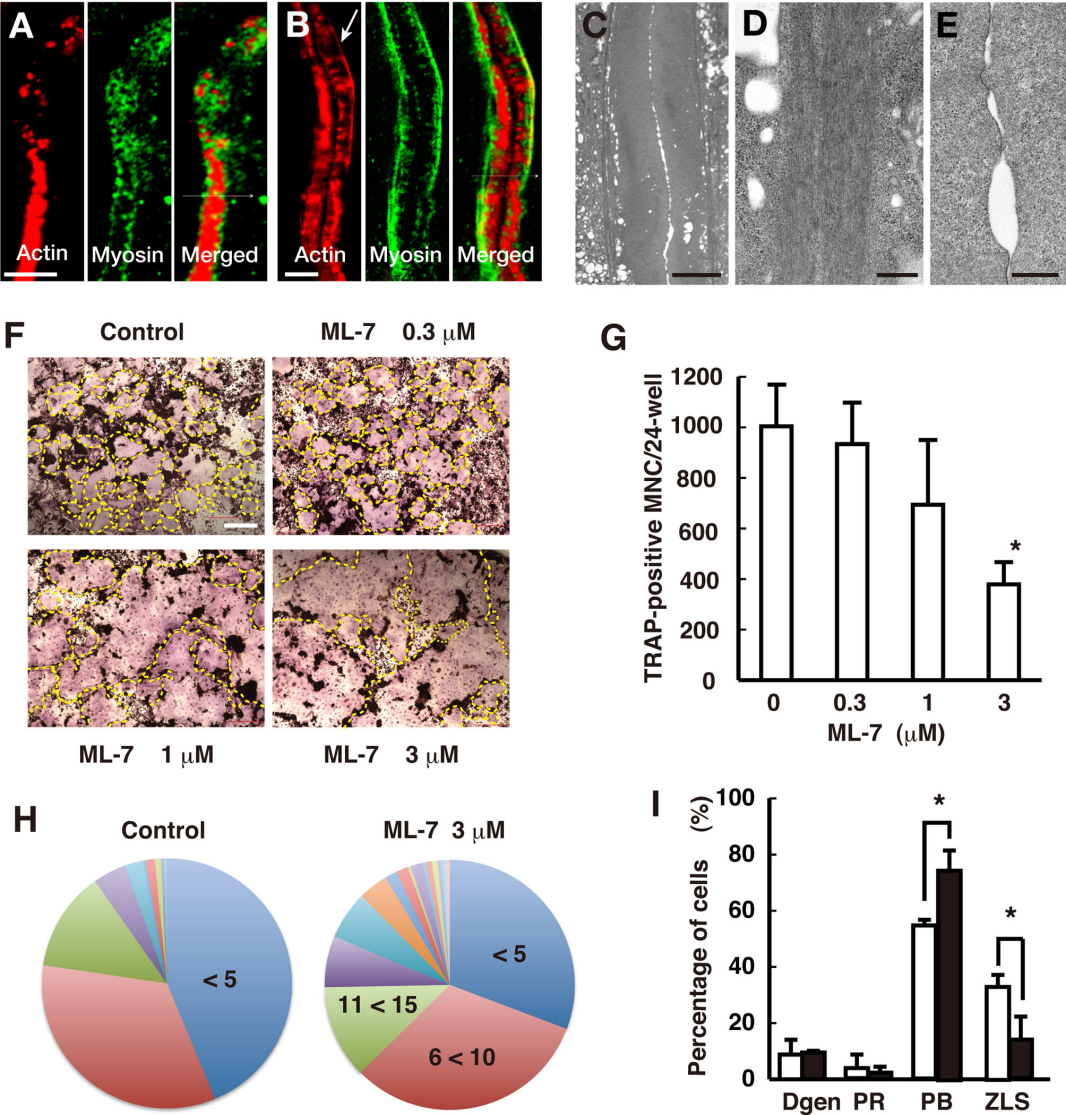
**Distribution of non-muscle myosin IIA in the ZLS and its role in osteoclast fusion.** Osteoclasts were stained with rhodamine-phalloidin (red) and an anti-non-muscle myosin IIA antibody (green). (A) Confocal image of the podosome belt. (B) Confocal image of the ZLS. The arrow indicates the line of F-actin that overlaps with non-muscle myosin IIA. (C) An image of the ZLS acquired via transmission electron microscopy. (D) Magnified view of the bundles running along the ZLS. Numerous parallel-running thin filaments were observed in the bundle. (E) Magnified view of the cell contact site in the ZLS. Thin filaments were rarely observed. (F) Images of TRAP-stained osteoclasts. The outline of an osteoclast is indicated by a yellow dashed line. (G) Effect of ML-7 on the formation of TRAP-positive multinucleated cells. Data are mean±s.d. (*n*=3). *Significantly different from the control, *P*<0.05, paired *t*-test. (H) Pie charts of the number of nuclei per osteoclast. Treatment with ML-7 (3 µM) resulted in larger osteoclasts than the control (control, *n*=256; ML-7, *n*=257; *P*<0.001, Mann–Whitney two-tailed test). (I) Effect of ML-7 on actin structures in osteoclasts. Osteoclastogenesis was performed in the absence (□) and presence (▪) of 3 µM ML-7. Fixed cells were stained with rhodamine-phalloidin. Osteoclasts were classified into four types: cells that had degenerated actin structures (Dgen); cells that had the podosome ring (PR); cells that had the podosome belt (PB); and cells that had the ZLS. More than 100 osteoclasts were counted per experiment. Data are mean±s.d. (*n*=3). *Significantly different from the control, *P*<0.05, paired *t*-test. Scale bars: 5 µm in A,B; 10 µm in C; 500 nm in D,E; 200 µm in F.

Osteoclasts express the contractile proteins non-muscle myosin IIA and IIB at their ventral membrane (Krits et al., 2002). To determine myosin distribution, we stained osteoclasts with an anti-non-muscle myosin IIA antibody and rhodamine-phalloidin, and imaged them by confocal microscopy. We found that although non-muscle myosin IIA surrounded the actin cores and was distributed on both sides of the podosome belt (Fig. 3A), as previously described (Ory et al., 2008), it was localized only on the inner side of the actin-rich region of the ZLS (Fig. 3B). Thus, the transition of the podosome belt into the ZLS appears to be accompanied by the polarized redistribution of non-muscle myosin IIA.

Because non-muscle myosin IIA alone cannot form long filaments, such as those observed in the ZLS, we asked whether an actin cable could be found in the ZLS, which functions as a scaffold for myosin. Careful examination revealed a line, positive for rhodamine-phalloidin, at the inner side of the actin-rich region of the ZLS (Fig. 3B, arrow). Transmission electron microscopy showed two thick electron-dense bands, extending along both sides of the actin-rich region of the ZLS (Fig. 3C). Each thick band consisted of many parallel-running filaments (Fig. 3D). In contrast, we did not detect any obvious filamentous structures in the actin-rich region, consistent with our previous findings (Fig. 3E). These results suggest that the stimulus upon cell-cell contact may cause F-actin rearrangements, resulting in the formation of F-actin bundles that likely recruit non muscle myosin IIA.

### The role of non-muscle myosin IIA in osteoclast fusion

To assess the role of non-muscle myosin IIA in osteoclast fusion, we examined the effect of ML-7, a myosin light chain kinase inhibitor, on osteoclastogenesis. Tartrate-resistant acid phosphatase (TRAP) staining of the cells showed that ML-7 treatment increased the size of differentiated osteoclasts (Fig. 3F). Although the number of osteoclasts formed in the presence of ML-7 was lower than that of the control, this was due to osteoclast hypermultinucleation and not to osteoclastogenesis inhibition (Fig. 3F,G). The average number of nuclei per cell increased from 8.43 to 13.4 in response to the treatment (Fig. 3H). ML-7 treatment decreased the percentage of osteoclasts that had fewer than five nuclei from 43.7% to 30.7%, whereas it increased the percentage of osteoclasts that had more than 20 nuclei from 9.7% to 25.2%. Thus, ML-7 promoted the formation of large osteoclasts, suggesting that the phosphorylation of non-muscle myosin II light chain is negatively involved in osteoclast fusion. ML-7 treatment had no discernible effect on the gross morphology of the ZLS (Fig. S2), suggesting the independence of myosin activity and F-actin bundle and ZLS formation. However, the treatment significantly increased the percentage of osteoclasts with a podosome belt, whereas it decreased the percentage of osteoclasts that possessed the ZLS (Fig. 3I), probably because it decreased the number of osteoclasts capable of forming the ZLS (Fig. 3G). Overall, inhibiting the activity of non-muscle myosin II promoted osteoclast hypermultinucleation, but it had little effect on the structure of the ZLS. This conclusion agrees with previous findings showing that non-muscle myosin IIA knockdown resulted in the formation of large osteoclasts and enhanced cell spreading (McMichael et al., 2009).

### Distribution of other podosomal proteins in the ZLS

**Fig. 4.**
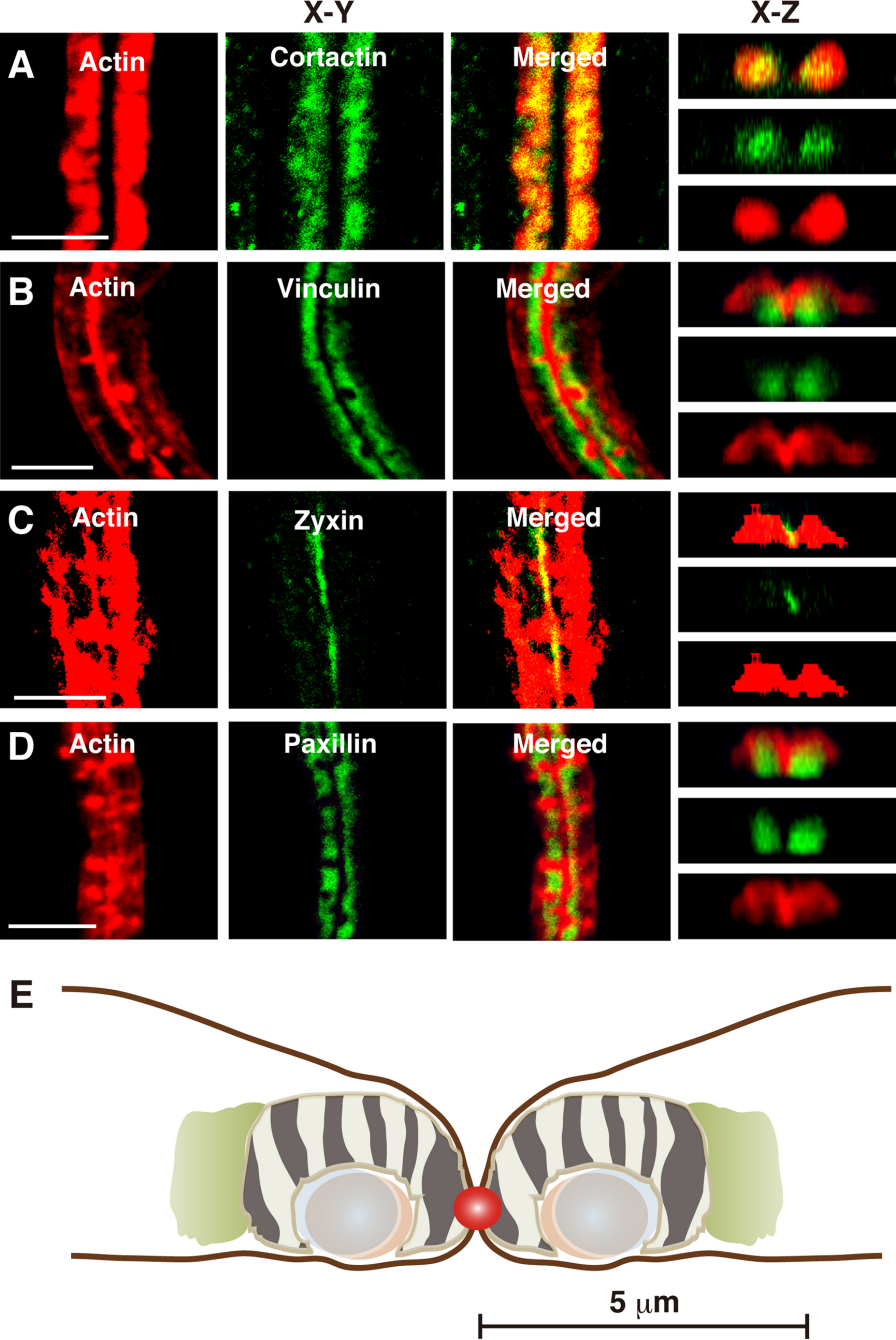
**Distribution of podosomal proteins in the ZLS.** Osteoclasts were stained with rhodamine-phalloidin and an anti-cortactin antibody (A), anti-vinculin antibody (B), anti-zyxin antibody (C) or anti-paxillin antibody (D), and imaged via confocal microscopy. Images shown are in the *xy*-plane and *xz*-axis. Scale bars: 10 µm. (E) Schematic representation of the ZLS. The image was generated based on measurements of the dimensions of each podosomal protein from confocal images, as described in the Materials and Methods. Data are presented in Table S1. F-actin, off-white; non-muscle myosin IIA, light green; paxillin, pale blue; vinculin, light brown; podosome-like dots and stripes, zebra pattern; zyxin, red. Scale bar: 5 µm.

To estimate the relative positions of podosomal proteins within actin-rich regions, fixed cells were doubly stained with rhodamine-phalloidin and an antibody against cortactin, vinculin, paxillin or zyxin, and imaged by confocal microscopy. The distribution for each protein was determined in the projected confocal images and is listed in Table S1. Cortactin, a lamellipodial protein that promotes branched F-actin elongation by recruiting Arp2/3, overlapped with actin throughout the actin-rich region (Fig. 4A). Vinculin, which is found in the adhesion domain of podosomes (Marchisio et al., 1988), and acts as a tension sensor in focal adhesions (Grashoff et al., 2010), localized in the lower part of the actin-rich region (Fig. 4B). Zyxin, another tension-sensitive protein involved in focal adhesions (Lele et al., 2006), s found in the adhesion domain of the podosome (Kaverina et al., 2003), localized in the center of the ZLS, probably at the contact site between the two osteoclasts (Fig. 4C). The small GTPase Rac1 displays a similar localization pattern (Takito et al., 2015). Paxillin, which is also localized at the adhesion domain in the podosome and is involved in podosome organization and matrix degradation by osteoclasts (Badowski et al., 2008), was found in the lower part of the ZLS and mostly overlapped with vinculin (Fig. 4D). These results suggest that the arrangement of podosomal proteins in the ZLS is different from that in its precursor, the podosome belt. The arrangement of podosomal proteins in the ZLS is schematically depicted in Fig. 4E. We have previously reported that vinculin and paxillin are distributed on the inner side of the actin-rich region of the ZLS in fusing osteoclasts, RAW 264.7 cells cultured for 3 days (Takito et al., 2012). In this study, we used RAW 264.7 cells cultured for 4 days. Therefore, although the gross morphology of F-actin appeared to be the same, the architecture of the adhesion domain of the ZLS differed between fusing and mature osteoclasts.

### Effects of CK-666 on ZLS and osteoclast fusion

**Fig. 5.**
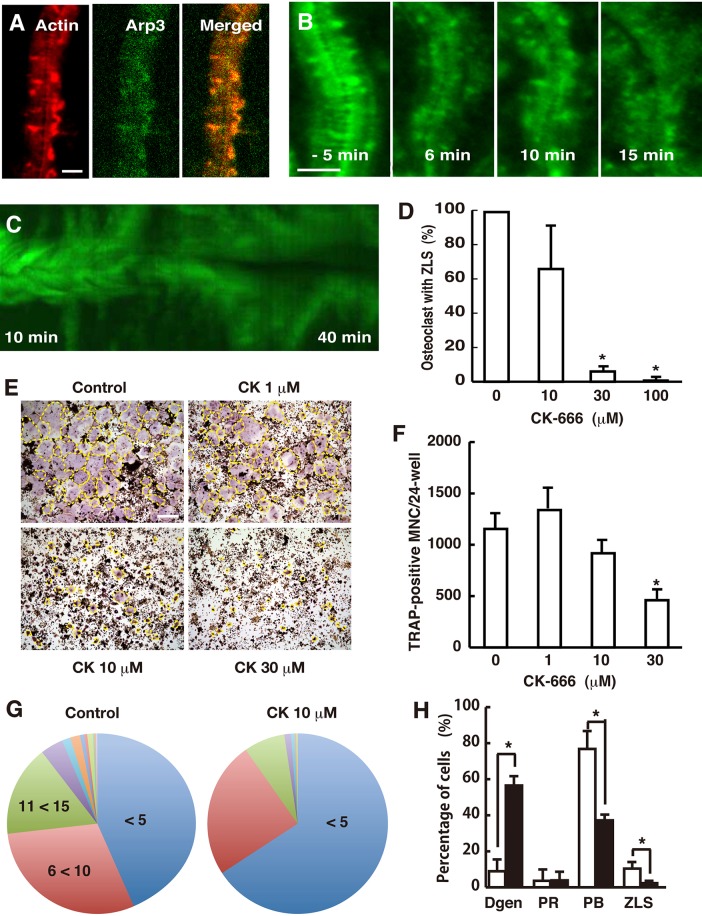
**Distribution of Arp2/3 in the ZLS and its role in osteoclast fusion.** (A) Osteoclasts were stained with rhodamine-phalloidin and an anti-Arp3 antibody. Scale bar: 5 µm. (B) Time-lapse images of the ZLS from cells treated with 100 µM CK-666. CK-666 was added at time 0. Scale bar: 5 µm. (C) A kymograph of EGFP-actin in the ZLS. The kymograph was generated from images taken 10 min after the addition of CK-666. (D) CK-666 disrupted the ZLS in a dose-dependent manner. Osteoclasts were incubated with the indicated concentration of CK-666 for 40 min. The cells were fixed, stained with rhodamine-phalloidin, and imaged by confocal microscopy. The number of osteoclasts that had the ZLS was counted on each slide. More than 100 osteoclasts were counted per experiment. The effect of CK-666 is expressed as the relative percentage of the control. Data are mean±s.d. (*n*=3). *Significantly different from the control, *P*<0.01, paired *t*-test. (E) Images of TRAP-stained osteoclasts. The outline of an osteoclast is indicated by a yellow dashed line. Scale bar: 200 µm. (F) Effect of CK-666 on the formation of TRAP-positive multinucleated cells. Data are mean±s.d. *n*=3. *Significantly different from the control, *P*<0.05, paired *t*-test. (G) Pie charts of the number of nuclei per osteoclast. Treatment with CK-666 at 10 µM resulted in smaller osteoclasts than the control (control, *n*=289; CK-666, *n*=217; *P*<0.001, Mann–Whitney two-tailed test). (H) Effect of CK-666 on the actin structures in osteoclasts. Osteoclastogenesis was performed in the absence (□) and presence (▪) of 10 µM CK-666. The fixed cells were stained with rhodamine-phalloidin. Osteoclasts were classified into four types: cells that had degenerated actin structures (Dgen); cells that had the podosome ring (PR); cells that had the podosome belt (PB); and cells that had the ZLS. More than 100 osteoclasts were counted per experiment. Data are mean±s.d. (*n*=3). *Significantly different from the control, *P*<0.05, paired *t*-test.

Arp2/3 regulates the branched elongation of actin filaments. The forces generated by Arp2/3-dependent retrograde actin flow in the leading lamellipodia push the membrane forward, resulting in cell migration (Pollard and Borisy, 2003). Arp2/3 localizes in the F-actin rich region of the ZLS (Fig. 5A) (Takito et al., 2012). We first tested the effect of CK-666, an Arp2/3 inhibitor, on ZLS actin dynamics. Live-cell imaging revealed that the organized array of F-actin in the ZLS was disturbed 6 min after the addition of 100 µM CK-666 (Fig. 5B). After 15 min, most F-actin stripes changed into podosome-like dots, and the ZLS lost its gross morphology. The kymographs indicate that the treatment caused the separation of the two connected osteoclasts (Fig. 5C). Next, osteoclasts were incubated with CK-666 for 40 min, fixed, stained with rhodamine-phalloidin, and imaged via confocal microscopy. CK-666 decreased the number of ZLS observed in a dose-dependent manner (Fig. 5D). The treatment also disrupted the normal cell adhesion between neighboring multinucleated cells (Fig. S3). These results suggest that the ZLS is an Arp2/3-dependent structure and is required for the juxtaposition of two osteoclasts.

We next examined the effect of CK-666 on osteoclastogenesis. CK-666 treatment decreased the size of differentiated osteoclasts, although its inhibitory effect on the number of generated osteoclast was minimal (Fig. 5E,F). Furthermore, it decreased the average number of nuclei per cell from 9.12 to 6.35 (Fig. 5G). These results suggest that Arp2/3-mediated actin branching activity positively influences osteoclast fusion. As expected, CK-666 (10 µM) decreased the percentage of osteoclasts containing a podosome belt and the ZLS, and increased the percentage of osteoclasts with degenerate actin structures (Fig. 5H). These results are consistent with those of a previous study showing that siRNA-mediated silencing of Arp2/3 in osteoclasts diminished the podosome belt (Hurst et al., 2004).

### Framework of the podosome field

Our results revealed that in cultured osteoclasts, EGFP-actin movement occurred frequently in all podosome-related structures (Fig. 1A-D). Notably, consistent symmetrical flow of EGFP-actin was observed throughout the ZLS width. These findings prompted us to reevaluate the structural framework of podosomes. The idea that the podosome cluster is an assembly of independent podosomes has been revised based on super-resolution microscopy observations, and is now considered as one multifunctional zone (van den Dries et al., 2013). This zone consists of three main substructures: actin cores, integrin islets, and a network of F-actin that connects the actin cores. Our findings add actin flow as a new element in this model. We denote this specified field, in which podosomes randomly assemble and disassemble (Evans et al., 2003), as the podosome field, which depends on four elements: actin cores, integrin islets, a network of F-actin, and actin flow.

### Evolution of the podosome field in osteoclasts

Self-organized traveling waves, driven by Arp2/3-dependent branched actin elongation (Carlsson, 2010), are involved in various cell functions (Allard and Mogilner, 2013). In *Dictyostelium discoideum* cells, traveling waves begin with local actin clustering, followed by the formation of a closed circle, with repeated expansion and retraction (Gerisch et al., 2011; Schroth-Diez et al., 2009). When traveling waves come to the cell periphery, they propel the membrane forward-forming broad lamellipodia (Bretschneider et al., 2009). The developmental pattern of traveling waves in *Dictyostelium* is similar to that of the podosome field in osteoclasts. Furthermore, the formation of waves is Arp2/3-dependent and independent of non-muscle myosin II (Bretschneider et al., 2009), similar to actin flow in the ZLS. On the basis of these observations, we propose that the evolution of this actin superstructure in osteoclasts depends on self-organized traveling waves in the podosome field, but not the podosomes, which represent another self-organized structure (Destaing et al., 2003). This unexpected conclusion is supported by the observation that gelsolin-deficient osteoclasts can form an actin belt in the absence of podosomes (Chellaiah et al., 2000). A recent study reported the presence of actin flow in the dendritic podosome cluster (Meddens et al., 2016), but the flow rate observed was <10% (0.1 µm/min) of our value and the flow was dependent on non-muscle myosin IIA. This discrepancy may be due to differences between the cell types or the state of the podosome field: podosome clusters versus the ZLS.

### The role of the ZLS in osteoclast fusion

**Fig. 6.**
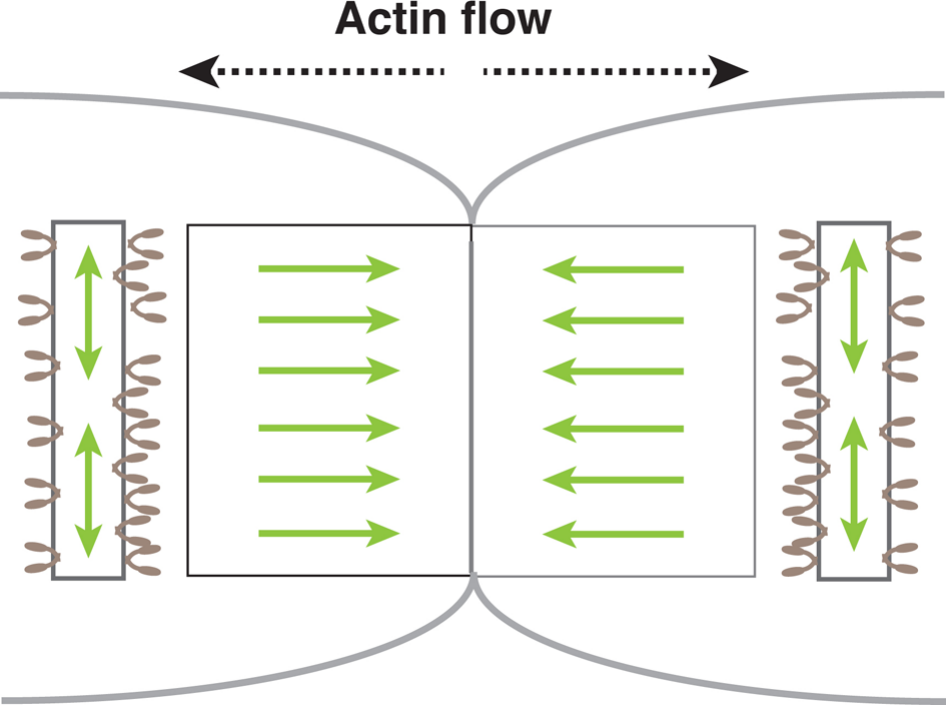
**A model of force balance around the ZLS.** Symmetrical retrograde actin flow generated by branched actin elongation via Arp2/3 in the ZLS generates forces that push the plasma membrane outward (light green left-right arrows) at the cell-cell contact site of the two osteoclasts. Two opposing forces result in the juxtaposition of the two cells in the ZLS. Non-muscle myosin IIA (light brown) bound to the circumferential linear actin bundle generates circumferential forces via actomyosin contraction (light green up-down arrows). The two forces may counteract each other and contribute to the adhesion between multinucleated cells during osteoclast fusion.

We previously proposed a role for the ZLS in cell-cell interactions during osteoclast fusion (Takito et al., 2012). Because E-cadherin is involved in osteoclast fusion (Mbalaviele et al., 1995), this cell adhesion molecule is expected to be present in the ZLS. However, our preliminary experiments showed that E-cadherin was not concentrated in the ZLS and confirmed previous observations of decreased E-cadherin mRNA at the fusion stage (Fiorino and Harrison, 2016; Yagi et al., 2005). Thus, how the ZLS promotes adhesion between the partner cells remains elusive. Symmetrical retrograde actin flow in the ZLS may be a driving force for cell-cell adhesion. As retrograde actin flow pushes the plasma membrane, symmetrical retrograde actin flow should maintain the juxtaposition of the two partner cells during cell fusion (Fig. 6). This idea is supported by our results showing that CK-666 disrupted cell adhesion and inhibited osteoclast hypermultinucleation (Fig. 5E-G). During multinucleated osteoclast fusion, there is a lag time of approximately 60 min between cell collision and membrane fusion, during which the partner cells form the ZLS at the cell-cell contact site (Takito and Nakamura, 2012; Takito et al., 2012). In contrast, fusion between mononuclear cells often is completed within a few minutes. Therefore, the ZLS may be the fusion structure that provides a mechanical scaffold to maintain the large contact area during fusion between large osteoclasts. Previous studies have proposed the involvement of a podosome-dependent protrusion structure in osteoclast fusion (Oikawa et al., 2012; Shin et al., 2014). This protrusion structure may also work as a mechanical scaffold in a distinct mode during osteoclast fusion.

### Antagonistic roles of actin flow and actomyosin contraction during osteoclast fusion

Actin stress fibers are expected to generate forces by actomyosin contraction. Electron microscopy observations revealed that long F-actin bundles run along both sides of the ZLS (Fig. 3C,D). The distribution of F-actin bundles overlapped with that of non-muscle myosin IIA (Fig. 3B). Such an arrangement of non-muscle myosin IIA and actin cables in the ZLS suggests that actomyosin contraction may produce circumferential forces that locally counterbalance the forces generated by the retrograde actin flow in the ZLS (Fig. 6). Consistent with this scenario, the osteoclasts formed in the presence of ML-7 were larger than the control cells (Fig. 3H). The function of non-muscle myosin IIA varies in the podosome field. Myosin is positively involved in the formation of podosomes in macrophages (Kopp et al., 2006) and the podosome rings in baby hamster kidney (BHK) cells transformed by Rous sarcoma virus (RSV) (Collin et al., 2008), whereas it is irrelevant for the formation of the podosome belt (McMichael et al., 2009) and the ZLS in osteoclasts (Fig. S2). These results indicate that the structure and local force balance in the podosome field are regulated by the relative arrangement of linear actin filaments against the direction of branched actin elongation. Future studies on how osteoclasts locally regulate and coordinate the two actin polymerization processes may shed new light on bone resorption.

## MATERIALS AND METHODS

### Materials

The following reagents were used: α-minimum essential medium, (α-MEM; 135-15175, WAKO, Osaka, Japan); fetal bovine serum (FBS; 10270, Gibco), anti-Arp3 antibody (sc-48344, 1:100, Santa Cruz Biotechnology); anti-β-actin antibody (ab8226, 1:100, Abcam); anti-non-muscle myosin heavy chain IIA antibody (PRB-440P-100, 1:100, BioLegend, Dedham, MA); anti-cortactin antibody [5-180, 1:100, Millipore (Upstate)]; anti-paxillin antibody (610051, 1:100, BD Biosciences); anti-vinculin antibody (V9264, 1:100, Sigma-Aldrich); anti-zyxin antibody (sc-6438, 1:100, Santa Cruz Biotechnology); rhodamine-phalloidin (R415, Molecular Probes, Thermo Fisher Scientific); fluorescein isothiocyanate-phalloidin (P5282, Sigma-Aldrich); DAPI (D1306, Molecular Probes, Thermo Fisher Scientific, CA); recombinant mouse receptor activator of nuclear factor kappa-Β (sRANK) ligand (315-11, PeproTech, NJ); fluorescein isothiocyanate- and tetramethylrhodamine isothiocyanate-conjugated secondary antibodies (715-095-151, 711-095-152 and 712-096-153, Jackson ImmunoResearch, PA); CK-666 (182515, Calbiochem, Merck Millipore, Darmstadt, Germany) and ML-7 (475880, Calbiochem).

### Osteoclastogenesis

Osteoclastogenesis was induced as previously described (Takito et al., 2012). Briefly, RAW 264.7 cells (ATCC TIB-71, American Type Culture Collection, Manassas, VA) were cultured in a 24-well culture dish (353047, Falcon, Durham, NC) at a density of 1×10^4^ cells per well in 0.5 ml α-MEM, supplemented with 10% FBS, antibiotic (15240-062, Gibco) and 100 ng/ml sRANKL. After 3 days in culture, the medium was replaced with fresh medium containing CK-666 or ML-7, and the cells were incubated for an additional 24 h. The control culture was treated with an equal volume of dimethyl sulfoxide (D2650, Sigma-Aldrich). For TRAP staining, the cells were stained using an Acid Phosphatase, Leukocyte (TRAP) Kit (387A-1KT, Sigma-Aldrich) according to the manufacturer's instructions. Osteoclasts were defined as cells with more than three nuclei. Osteoclast formation was quantified by averaging the number of TRAP-positive multinucleated cells from three wells. Data from three independent experiments were collected.

### Counting the number of nuclei in osteoclasts

RAW 264.7 cells were differentiated into osteoclasts on a cover glass (12-545-82, Fisher Scientific), in a 24-well culture dish as previously described (Takito et al., 2015). After 3 days in culture, the medium was replaced with fresh medium containing CK-666 or ML-7, and the cells were incubated for an additional 24 h. The control culture was treated with an equal volume of dimethyl sulfoxide. For counting the number of nuclei in osteoclasts, the cells were fixed, stained with rhodamine-phalloidin and DAPI, and imaged via confocal microscopy. Osteoclasts were defined as cells with more than three nuclei. More than 100 osteoclasts were counted per experiment. Data from three independent experiments were collected and histograms with a bin-width of 5 were constructed using Microsoft Excel. The distributions are presented as pie charts.

### Immunostaining

RAW 264.7 cells were cultured on a glass coverslip in a 24-well culture dish for 4 days in the presence of sRANKL. The culture medium was changed every 2 days. The cells were fixed with 4% paraformaldehyde in phosphate-buffered saline (PBS) for 30 min, washed twice with PBS, permeabilized with 0.5% NP-40 (492016, Calbiochem) in PBS for 5 min and incubated with 1% bovine serum albumin (A7906, Sigma-Aldrich) in PBS for 1 h. The cells were then incubated with the primary antibody (non-muscle myosin IIA, cortactin, vinculin, paxillin or zyxin) in 1% bovine serum albumin in PBS at 4°C for 16 h, washed twice with PBS, and incubated with the appropriate secondary antibodies and rhodamine-phalloidin in 0.1% bovine serum albumin in PBS for 30 min. The stained cells were embedded with VECTASHIELD Antifade Mounting Medium (H-1000, Vector Laboratories, Burlingame, CA). Confocal imaging was performed on a Nikon A1si microscope equipped with a 60×/1.3 NA oil objective lens (Nikon, Tokyo, Japan). 3D images of the ZLS were reconstructed from *z*-section images acquired at 0.3 or 0.4 µm intervals. The nominal optical resolution was 0.3 µm and 0.49 µm along the *xy*-axis and *z*-axis, respectively. Nikon NIS-Elements AR-3.0 software was used for image analysis. The images shown were adjusted for contrast and brightness using Adobe Photoshop CS.

### N-SIM

Super-resolution images were acquired with a Nikon structured illumination microscope equipped with a 100×/1.49 NA oil objective lens. The stained cells were embedded with ProLong Diamond Antifade Mountant (P36965, Molecular Probes, Eugene, OR). The nominal optical resolution was 0.15 µm and 0.3 µm along the *xy*-axis and *z*-axis, respectively. The acquired images were analyzed with Nikon NIS-Elements AR-3.0 software.

### Contour map of the ZLS

To construct a contour map of the podosomal proteins in the ZLS, fixed cells were doubly stained with rhodamine-phalloidin and an antibody against non-muscle myosin IIA, cortactin, vinculin, paxillin or zyxin, and imaged via confocal microscopy. The projected image of the ZLS was reconstructed from *z*-section images once per osteoclast. The maximum contour of F-actin and each podosomal protein was measured at five points along the length of the ZLS in the projected image and averaged. Images were acquired from 11-65 osteoclasts in three independent experiments. Fig. 4E was drawn using Adobe Illustrator CS5.

### Height measurements

Cells were stained with rhodamine-phalloidin. *z*-stacks of each actin superstructure encompassing the entire Rhodamine-positive volume were acquired at 0.5 µm (confocal microscopy) or 0.3 µm (N-SIM) intervals. The average height of the podosome ring, the podosome belt and the ZLS was calculated from measurements in four points per projected image. The maximum height of each podosome was measured once in the projected image of the podosome cluster. Confocal microscopy images were acquired from three independent experiments and N-SIM images from two independent experiments.

### Measurement of the distance between the actin cores

Cells were stained with rhodamine-phalloidin. The distance between the actin cores was determined from the projected confocal images. The interpodosomal distance was defined as the center-to-center distance between the actin cores in the podosome cluster. The distance between actin cores in the ZLS was defined as the peak-to-peak distance in the surface intensity profile created using Nikon NIS-elements software (Fig. 2D). Images were acquired from three independent experiments.

### Live-cell imaging

RAW 264.7 cells were plated on a 35-mm glass-bottomed culture dish (P35G-1.5-10-C, MatTek, Ashland, MA; 3910-035-NYP, Iwaki, Asahi Glass, Tokyo, Japan), and transfected with EGFP-actin (6116-1, Clontech, CA) using FuGENE HD transfection reagent (E231A, Promega, Madison, WI) according to the manufacturer's instructions. The cells were then cultured in the presence of sRANKL for 4 days. Immediately before imaging, the culture medium was again supplemented with 100 ng/ml sRANKL. Time-lapse confocal images of EGFP-actin in osteoclasts were acquired with an Olympus FV10i microscope (Olympus, Tokyo, Japan) equipped with a 60× water objective lens in a 5% CO_2_ humidified atmosphere at 37°C, every 4 s for at least 13 min. Imaging at 1 s intervals yielded similar results. The focal width of the confocal scanning was set to 2.0 µm. Kymographs were generated from the time series images using ImageJ (https://imagej.nih.gov/ij/). The actin flow rate was calculated by identifying the trajectory of the EGFP-positive spots on the kymograph, as shown in Fig. 1A. Data were collected from 17 independent experiments. For the inhibitor (CK-666) experiments, recording was performed on a Nikon A1si microscope using a 60×/1.4 NA oil objective lens at room temperature. The cells were imaged for 10 min before the addition of CK-666 and for 40 min thereafter.

### Electron microscopy

Transmission electron microscopy was performed with a JEM-1200EX microscope (JOEL, Tokyo, Japan) operating at 80 kV, as previously described (Takito et al., 2012).
